# The Predictive Value of Carotid Artery Strain and Strain-Rate in Assessing the 3-Year Risk for Stroke and Acute Coronary Syndrome in Patients with Metabolic Syndrome

**DOI:** 10.31083/j.rcm2304146

**Published:** 2022-04-13

**Authors:** Sergiu Florin Arnăutu, Vlad Ioan Morariu, Diana Aurora Arnăutu, Mirela Cleopatra Tomescu

**Affiliations:** ^1^Neurology Department, Victor Babeș University of Medicine and Pharmacy, 300041 Timișoara, Romania; ^2^Multidisciplinary Heart Research Center, Victor Babes University of Medicine and Pharmacy, 300040 Timisoara, Romania; ^3^Cardiology Clinic, Timisoara Municipal Clinical Emergency Hospital, 300040 Timișoara, Romania

**Keywords:** major cardiovascular events, metabolic syndrome, carotid strain, carotid strain rate, subclinical atherosclerosis

## Abstract

**Purpose:**

The goal of this study is to see if carotid strain and strain rate can predict major cardio-vascular events (MACE) in people who have metabolic syndrome (MS) over a 3-year period of time.

**Methods:**

In this prospective observational research, we enrolled 220 adult MS patients (60.7 ± 7.5 years old, 53% male). Two-dimensional common carotid carotid artery (CCA) speckle-tracking ultrasound was used to determine the peak circumferential strain (CS) and the peak circumferential strain rate (CSR). Clinical outcomes were assessed throughout a three-year follow-up period.

**Results:**

After a 3-year follow-up period follow-up, 14 (7%) experienced MACE: Eight (4%) suffered an atherothrombotic ischemic stroke, four (2%) had acute coronary syndrome, and two (1%) were hospitalized for heart failure. Univariate regression analysis of the clinical and echocardiographic features of the MS patients found that age, systemic hypertension, diabetes mellitus, and the CCA circumferential strain and strain rate were significantly associated with the risk of MACE. Multivariate logistic regression identified two independent predictors of MACE in patients with MS, namely the CCA-related CS (%) and CSR (1/s), *p *< 0.01. The Receiver operating characteristic (ROC) curve analyses of these independent predictors of MACE indicated appropriate sensitivities and specificities. CS (AUC = 0.806, sensitivity = 82.6%, specificity = 79.2%, *p *< 0.0001) and CSR (AUC = 0.779, sensitivity = 82.6%, specificity = 72.4%, *p *< 0.0001) with cut-off values of ≤2.9% for carotid CS and ≤0.35 $s^{-1}$ for carotid CSR. Using these cut-off values, we obtained Kaplan-Meier survival curves, and these showed that MACE, ischemic stroke, and ACS-free survival was significantly lower among the MS patients with lower carotid CS and CSR (*p *< 0.0001).

**Conclusions:**

Carotid CS and CSR were independent predictors of major cardio- and cerebro-vascular events in prospectively monitored MS patients without established cardiovascular disease. Carotid deformation could be valuable as an early prognostic indicator for the cardiovascular risk in this population group.

## 1. Introduction

The metabolic syndrome (MS) is a well-known disorder characterized by the coexistence of a number of cardiovascular risk factors, including dyslipidemia, abdominal obesity, hyperglycemia, insulin resistance, and hypertension. It is more prevalent in sedentary and obese individuals and is associated with an increased risk of stroke, diabetes, myocardial infarction, and heart failure [1, 2, 3, 4]. Its prevalence has increased in recent years, jeopardizing the general population’s health. MS affects around 25% of adult individuals [1]. According to certain clinical studies, MS is connected with the presence of vascular atherosclerotic lesions [5]. The development of metabolic syndrome and cardiovascular events associated this disorder is expected to increase in the future years [6]. MS is a multifactorial illness that manifests itself via endothelial dysfunction, insulin resistance, diabetes, hypertension, abdominal obesity, dyslipidemia, and an elevated risk of atherothrombotic vascular events. It is mostly caused by an imbalanced diet, poor socioeconomic and cultural status, stress, and a sedentary lifestyle [7, 8, 9, 10].

Reduced arterial elasticity is an indicator of functional changes caused by atherosclerosis in the regional arterial system, including the carotid arteries. Speckle tracking echocardiography (STE) is a method for detecting cardiac deformation that has also been used to examine carotid artery vascular wall deformation. Catalano *et al*. [11] showed that the circumferential strain (CS) and circumferential strain rate (CSR) of the common carotid artery (CCA) represent a sensitive way of determining arterial rigidity. Another study demonstrated a relationship between carotid CS and CSR and previous stroke in elderly patients [12]. However, the prognostic value of carotid arterial strain in people with MS has not yet been investigated.

In this study, we used STE to quantify the CCA strain and strain rate in MS patients in order to establish their predictive value for MACE over a three-year follow-up period.

## 2. Material and Methods

### 2.1 Subject Selection 

This is a prospective observational study in which MS adult and older patients hospitalized sequentially in the Cardiology Department of the Timisoara Clinical Emergency Municipal Hospital (Timisoara, Romania) between November 2015 and November 2018 were investigated and monitored for MACE (acute coronary syndromes, ischemic atherothrombotic stroke, hospitalization for heart failure, and cardio-vascular death) for three years after discharge.

In order to be enrolled in the study, the patients had to be aged between 40 and 70 years old, have a verified diagnosis of metabolic syndrome, and have the ability to consent.

Refusal to provide informed consent, congenital vascular abnormalities, history of stroke or transient ischemic attacks, established cardiovascular disease, atrial fibrillation or flutter, life-threatening illness or cancer, and pregnancy or lactation were all considered exclusion criteria.

### 2.2 Data Extraction

Baseline demographic and clinical data, laboratory data, 12-lead resting electrocardiogram, echocardiographic data, and medical history were acquired from hospital records. After participants had fasted for more than 12 hours, blood samples from the peripheral venous system were taken in the morning. Using normal protocols, we determined the blood cell count, hemoglobin, electrolytes, cholesterol, triglycerides, glucose, glycated hemoglobin (HbA1c), and creatinine levels in centrifuged blood samples. At admission, the estimated glomerular filtration rate was computed using a simplified Modification of Diet in Renal Disease formula [13]. At discharge, medical treatment records were completed.

### 2.3 Definition of Covariates

MS was defined as the presence of any 3 of the following 5 risk factors: Abdominal obesity (waist circumference ≥80 cm for women and ≥94 cm for men); increased fasting plasma glucose (FPG) of ≥100 mg/dL or treatment of earlier diagnosed type two diabetes; increased triglyceride level of ≥150 mg/dL or medication for this lipid disorder; decreased high-density lipoprotein cholesterol of <40/50 mg/dL (men/women); systolic blood pressure (SBP) of ≥130 or diastolic blood pressure (DBP) of ≥85 mmHg or medication of earlier detected hypertension [14].

Diabetes mellitus was diagnosed when the patient’s fasting plasma glucose concentration was ≥126 mg/dL on two separate occasions or when on insulin or oral hypoglycemic treatment [14].

MACE included acute atherothrombotic ischemic stroke (AIS), acute coronary syndromes (ACS), hospitalization for heart failure (HF), and all-cause death.

Acute IAS was diagnosed in the presence of clinical signs of cerebral dysfunction (confirmed by neurological examination) lasting more than 24 hours, and associated with brain imaging evidence of infarction [15].

ACS was diagnosed in individuals without ST-segment elevation who had ischemic symptoms lasting more than 20 minutes at rest over the preceding 24 hours and either troponin or creatine kinase-MB exceeding the local lab-specific upper limit of normal or a positive bedside troponin assay [16]. ACS was diagnosed in patients with ST-segment elevation who had ischemic symptoms lasting at least 20 minutes at rest within the preceding 24 hours and at least one of the subsequent settings: persistent ST-segment elevation greater than 1 mm in >2 adjacent electrocardiographic leads, new or supposed new left bundle-branch block, or solitary posterior myocardial infarction [17].

### 2.4 Conventional Ultrasound Evaluation of the Heart and the Common Carotid Artery 

The same investigator conducted conventional echocardiography utilizing a VIVID5S, G.E. phased array ultrasonoscope (Tirat Carmel, Israel) equipped with a 3.5 MHz transducer. The heart chamber sizes were determined in accordance with the American Society of Echocardiography’s recommendations [18]. The systolic left ventricular function was assessed by measuring left ventricular ejection fraction (LVEF) using Simpson’s biplane method. The left atrial volume index (LAVI) to body surface area was calculated in the apical view, using the biplane area length method. The total left atrial emptying fraction (LAEF) was calculated using the formula: (LAEDV-LAESV)/LAEDV × 100.

Bilateral common carotid artery (CCA) B-mode images were obtained with the same ultrasonoscope using a multifrequency 7.5–10 MHz high resolution vascular probe [19]. The probe was held perpendicular to the CCA far wall. For three cardiac cycles, bilateral long-axis images of the common carotid arteries up to the carotid bulb were taken to enhance visibility of the intima-media system. Sinus rhythm was used to gather images, and premature beats were excluded. Carotid IMT (intima-media thickness) was measured at the end of diastole in the CCA, 1.0 cm proximal to the carotid bulb, and plaque presence was identified when there was IMT ≥15 mm [20, 21]. Peak systolic flow velocity, end-diastolic flow velocity, pulsatility index, and resistance index of the common carotid artery were measured using Doppler ultrasonography.

### 2.5 2D-STE Evaluation of the Carotid Artery 

Cross-sectional pictures of the common CCA were obtained for three cardiac cycles at a frame rate of 60–90 frames per second. The images were all saved digitally in RawDICOM mode, and exported from the ultrasound equipment and then analyzed offline with the EchoPAC software version 2011 (GE-VingMed, Horten, Norway). Using a point-and-click approach, the inner vascular margin was manually delineated. The zone of investigation was carefully tailored to include the whole thickness of the vessel wall. The adequate tracking of the vascular walls was verified visually and automatically. The system separated the overall carotid artery circumference into 6 standard segments and assigned each segment a tracking quality. If the tracking was unsatisfactory, the operator could improve its quality by either changing the width of the focused region or by adjusting the endothelium lining. If all six segments had acceptable tracking quality, the EchoPAC software automatically calculated the CS and the CSR of CCA.

To prevent overcoming deformations within a pulsation, we did not utilize the compound setting to smooth the tracking. Each ultrasonographic picture was obtained by the same operator who was unaware of the subject’s clinical features. In our lab, ten random samples of the carotid artery were used to check the consistency of speckle tracking.

The reliability of speckle tracking for carotid artery was evaluated using ten random samples. For CS, the intraobserver concordance correlation coefficient (ρ) was 0.94, and the intraobserver agreement statistic (K) was 0.77; the interrater ρ was 0.93, and the interrater K was 0.70. For CSR, the intraobserver ρ was 0.94 and the intraobserver K was 0.74, while the interobserver ρ was 0.92 and the interobserver K was 0.74. Carotid deformation reliability in our research was equivalent to that seen in another investigation [11].

### 2.6 Follow-Up and Outcome

Patients and their families were advised to inform the attending physician as soon as possible if they had any health issues that required emergency medical treatment or hospitalization. Every 6 months following discharge, patients were contacted over the phone to give information on their health status. In the event of hospital admissions, further information was gathered from medical records.

### 2.7 Statistical Analysis 

Unless otherwise noted, all data is presented as mean standard deviation. Correlations between categorical and continuous variables were determined using the chi-square test for categorical variables and the independent-samples *t*-test for continuous variables. Multivariate regression analysis was utilized to discover independent predictors of cardiovascular and cerebrovascular end points. Odds ratios (ORs) and 95% confidence intervals (CIs) were calculated the using Cox proportional hazards models. Receiver operating characteristic (ROC) curves were used to determine appropriate cut-off values for the independent predictors of the end-points. The threshold for statistical significance was established at a *p*-value of <0.05 using the MedCalc Statistical Software version 20.15 (MedCalc Software Ltd, Ostend, Belgium) for Windows.

## 3. Results

A total of 235 MS patients were registered in the present analysis. Fifteen patients were eliminated because of the unacceptable quality of echocardiographic images. Finally, 220 MS patients were enrolled in the study. Their mean age was 60.7 ± 7.5 years, and 118 (53%) were men. MACE occurred in 14 MS patients (7%), as follows: eight (4%) atherothrombotic ischemic strokes, six (2%) ACS, and two (1%) were hospitalized for HF. According to whether they had suffered a MACE during the 3-year follow-up, the MS patients were divided into two groups. Baseline demographics, clinical characteristics, and echocardiographic features of the MS patients are shown in Tables 1,2.

**Table 1.** **Baseline clinical and biochemical characteristics of MS patients**.

	With MACE (n = 14)	Without MACE (n = 206)	*p* value
Age (Years)	64.0 ± 6.2	59.7 ± 6.7	**0.02**
Male sex	5 (35%)	113 (55%)	0.14
Systemic hypertension (n, %)	11 (80%)	103 (50%)	**0.03**
Diabetes mellitus	106 (69%)	97 (24%)	< **0.001**
Smoking (current, %)	2 (10%)	25 (12%)	0.82
Systolic BP (mmHg)	151.5 ± 17	131.27 ± 12	< **0.0001**
Diastolic BP (mmHg)	84.6 ± 11	73.23 ± 6.97	< **0.0001**
Heart rate (beats/min)	75.6 ± 11.4	73.11 ± 10.8	0.40
BMI (kg/$m^{2}$)	32.7 ± 5.2	31.7 ± 3.8	0.35
Waist circumference (cm)	101 ± 7	99 ± 10	0.46
HDL-C (mg/dL)	46 ± 13	48 ± 12	0.54
LDL-C (mg/dL)	197 ± 44	184 ± 39	0.23
Triglyceride (mg/dL)	159.1 ± 89.5	134.4 ± 80.4	0.16
FPG (mg/dL)	110.5 ± 32	109.4 ± 33	0.90
HbA1c	6.3 ± 0.01	6.1 ± 0.02	< **0.0001**
ASAT	24 ± 9	23 ± 5	0.49
ALAT	37 ± 7	36 ± 5	0.48
NT-pro BNP	97 ± 19	96 ± 15	0.61
ACEI	4 (30%)	76 (37%)	0.59
ARB	7 (50%)	72 (35%)	0.25
CCB	10 (72%)	155(75%)	0.80
Diuretics	4 (28%)	48 (23%)	0.67
Beta-blocker	4 (27%)	60 (29%)	0.87
Oral antidiabetics	3 (23%)	20 (10%)	0.13
Insuline	1 (7%)	3 (1%)	0.37

Notes: Data are expressed as mean ± SD or number (percentage). Statistically significant values are highlighted in bold (*p *< 0.05). Abbreviations: MS, metabolic syndrome; BMI, body mass index; BP, blood pressure; BMI, body mass index; HDL, high density lipoprotein; LDL, low density lipoprotein; FPG, fasting plasma glucose; HbA1c, glycosylated hemoglobin; ASAT, aspartat amino transferase; ALAT, alanine amino transferase; NT-pro BNP, N-type brain natriuretic peptide; ACEI, angiotensin-converting enzyme inhibitors; ARB, angiotensin II receptor blocker; CCB, calcium channel blocker.

**Table 2.** **Comparison of carotid and cardiac parameters in MS and control groups**.

	With MACE (n = 14)	Without MACE (n = 206)	*p* value
LVEF (%)	62.7 ± 2.6	63.8 ± 3.2	0.21
LVFS (%)	36.93 ± 2.91	38.00 ± 3.50	0.26
LA index volume (mL/$m^{2}$)	27.3 ± 5.2	26.6 ± 5.7	0.65
LA ejection fraction (%)	58.2 ± 4.0	57.8 ± 3.4	0.67
IMT (mm)	0.88 ± 0.22	0.81 ± 0.18	0.16
Carotid plaque present n (%)	17 (74%)	142 (72%)	0.86
DD diameter (mm)	6.28 ± 0.94	6.22 ± 1.04	0.83
SD diameter (mm)	6.78 ± 0.91	6. 68 ± 1.06	0.73
PSV (cm/s)	67.74 ± 12.26	70.48 ± 9.90	0.32
EDV (cm/s)	18.74 ± 4.08	18.67 ± 3.70	0.94
PI	1.13 ± 0.07	1.16 ± 0.09	0.22
RI	0.72 ± 0.04	0.74 ± 0.06	0.22
CCA CS (%)	2.12 ± 0.7	3.33 ± 1.14	**0.0001**
CCA CSR ($s^{-1}$)	0.31 ± 0.11	0.46 ± 0.13	< **0.0001**

Notes: Data are expressed as mean ± SD or number (percentage). Statistically significant values are highlighted in bold (*p *< 0.05). Abbreviations: MS, metabolic syndrome; LVEF, left ventricular ejection fraction; LVFS, left ventricular fractional shortening; LA, left atrium; IMT, intima media thickness; DS, end systolic diameter; DD, end diastolic diameter; PSV, peak systolic velocity; EDV, end diastolic velocity; PI, pulsatility index; RI, resistivity index; CCA, common carotid artery; CS, circumferential strain; CSR, circumferential strain rate.

The group I patients were more frequently older, hypertensive, and diabetic. The echocardiographic conventional ultrasound assessment of the left and right CCA showed no differences among the MS patients. Significant differences were noted among the two groups regarding the CCA strain parameters: CS (2.12 ± 0.7% vs 3.33 ± 1.14%, *p *< 0.0001) and CSR (0.31 ± 0.11 $s^{-1}$ vs 0.31 ± 0.1 $s^{-1}$, *p <* 0.0001) (Table 2).

Using univariate regression, we found that age, systemic hypertension, diabetes mellitus, and the circumferential strain and strain rate of the CCA were all linked to a higher risk of MACE in people with MS (Table 3).

**Table 3.** **Predictors of MACE in MS patients**.

Parameter	Univariable OR (95% CI)	*p*-value	Multivariable OR (95% CI)	*p*-value
Age (years)	1.12 (1.03–1.22)	< **0.01**		
HTN	1.02 (1.00–1.04	**0.03**		
DM	0.63 (0.25–1.56)	< **0.01**		
CCA-CS (%)	0.11 (0.04–0.25)	< **0.0001**	1.04 (0.01–0.32)	< **0.01**
CCA-CSR (1/s)	1.71 (2.05–13.3)	< **0.0001**	0.43 (0.00–0.7)	< **0.01**

Note: Statistically significant values are shown in bold (*p *< 0.05) Abbreviations OR, Odds ratio; HTN, systemic hypertension; DM, diabetes mellitus; CCA, common carotid artery; CS, circumferential strain; CSR, circumferential strain rate.

Multivariate logistic regression included the predictors of MACE identified by univariate analysis and found two independent predictors of MACE in patients with MS, namely the CCA-related CS (%) and CSR (1/s), *p *< 0.01, (Table 3).

The ROC curve analyses of these independent predictors of MACE indicated appropriate sensitivities and specificities. CSR (AUC = 0.779, sensitivity = 82.6%, specificity = 72.4%, *p *< 0.0001); CS (AUC = 0.806, sensitivity = 82.6%, specificity = 79.2%, *p *< 0.0001).

**Fig. 1.**
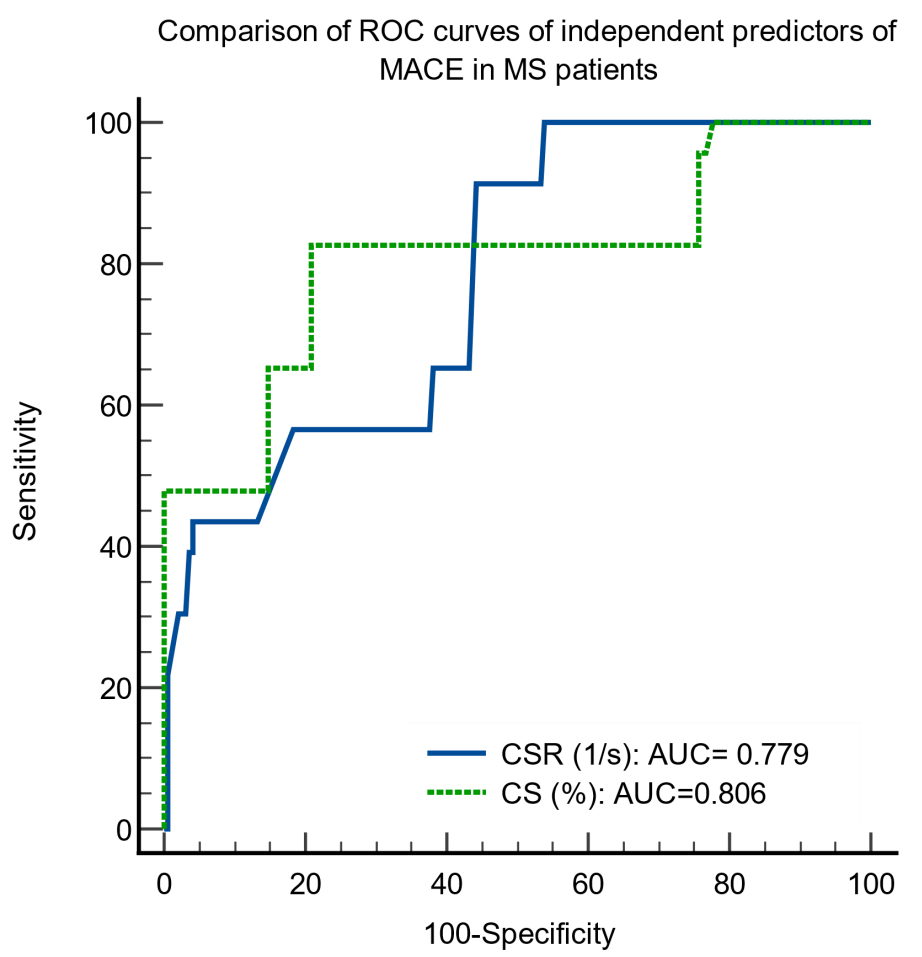
**Comparison of ROC curves of independent predictors of major cardiovascular events in metabolic syndrome patients**. ROC, receiver operating curves; MACE, major cardiovascular events; MS, metabolic syndrome; CSR, circumferential strain rate; CS, circumferential strain.

The comparison of the ROC curves showed no significant differences between the areas under the ROC curves for CS and CSR (Fig. 1), *p* = 0.67.

The identified cut-off values for the independent predictors of MACE by the ROC curves were CS ≤2.9%, and CSR <0.35 $s^{-1}$.

**Fig. 2.**
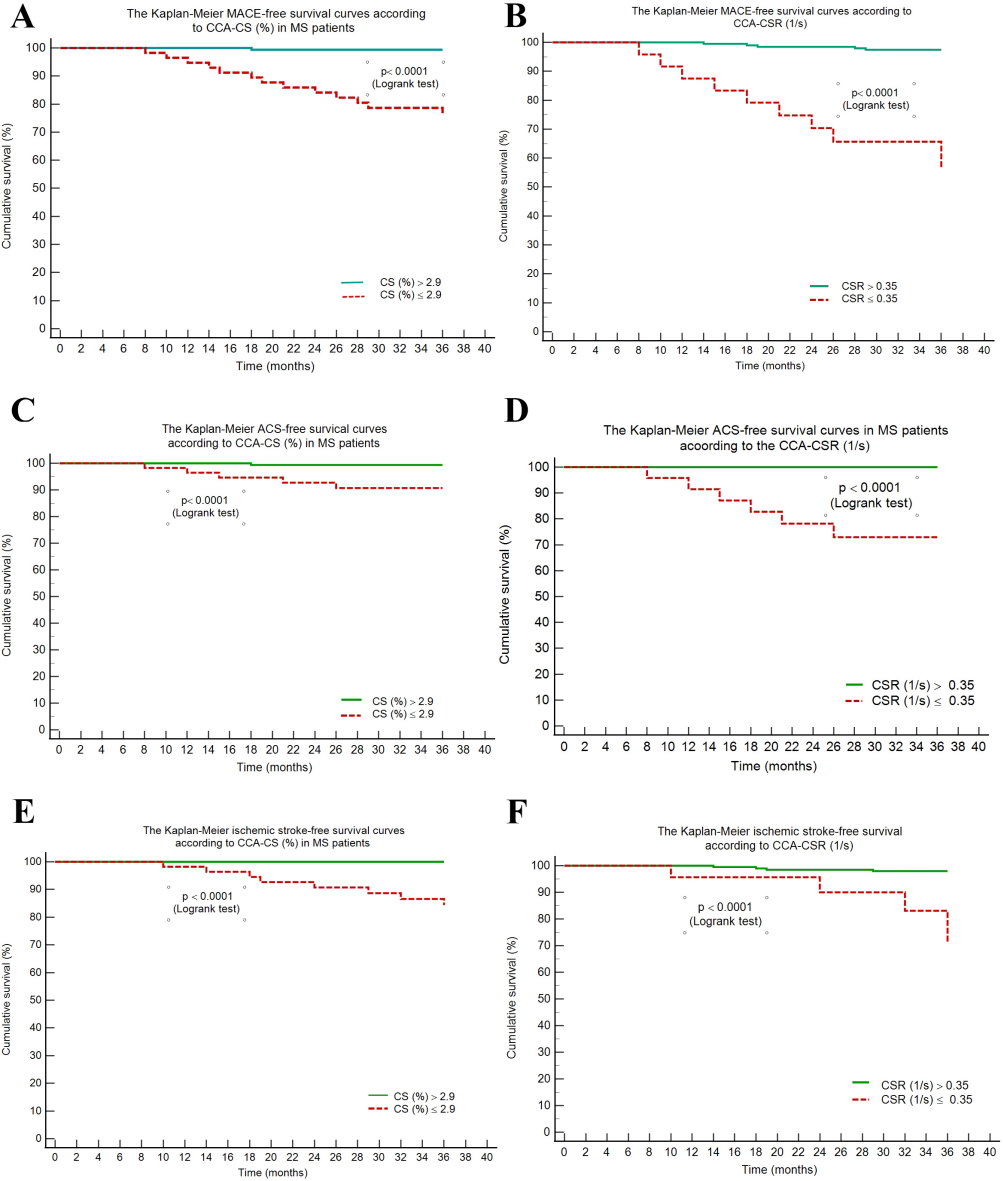
**The Kaplan-Meier MACE-free survival curves in metabolic syndrome patients according to common carotid artery circumferential strain and strain rate rate**. MACE, major cardiovascular events; MS, metabolic syndrome; CCA, common carotid artery; ACS, acute coronary syndrome; CS, circumferential strain; CSR, circumferential strain rate.

Using these cut-off values, we obtained Kaplan-Meier survival curves, and these showed that MACE, ischemic stroke, and ACS-free survival was significantly longer among the MS patients with higher CS and CSR (*p *< 0.0001), as shown in Fig. 2.

## 4. Discussion

To our awareness, this is the first study that examined the carotid artery strain and strain rate to see if they could predict major cardiovascular events in people with MS.

We established that there is a strong connection between the risk of vascular atherothrombotic events and reduced carotid wall deformation patterns in MS patients. This association was independent of the presence of carotid plaque and IMT, as well as of blood pressure-lowering drugs that may have had an influence on arterial wall function. Our findings suggest that reduced CCA circumferential strain and strain rate represent an early indicator of subclinical atherosclerosis and are linked with an increased risk of vascular events.

Functional deterioration of the artery wall may develop early in the atherosclerotic process, prior to the appearance of structural wall alterations, such as atherosclerotic plaque or elevated IMT, and may precede the onset of clinical symptoms [22]. Earlier studies proved that individuals with MS and endothelial dysfunction demonstrated by brachial artery flow-mediated dilatation had an augmented cardiovascular risk than those with only one of these disorders [23].

Carotid ultrasonography is a noninvasive method for assessing carotid artery atherosclerosis and may be used in addition with established risk scores to estimate the risk of cardiovascular events. Although carotid IMT is traditionally utilized, the existing data suggests that its prognostic value is variable [24]. A large collaborative research found that carotid IMT had little predictive value for cardiovascular risk in people with systemic hypertension [25]. The European Lacidipine Study on Atherosclerosis (ELSA) showed that alterations in carotid IMT after therapy did not influence the outcomes in individuals with hypertension [26]. In the elderly, researchers previously established that stroke is linked with carotid strain and strain rate but not with carotid IMT [12]. A potential explanation for the weaker association between IMT and cardiovascular events is that carotid IMT is measured on a very small interval band, and changes in IMT after therapy are even finer [27]. Additionally, IMT only detects morphological and structural alterations in carotid arteries, while carotid 2D-STE evaluates the whole carotid artery wall in a functional, contractile approach. Carotid CS and CSR are therefore important markers of the carotid artery’s local characteristics and function [11, 28]. Traditional indicators of arterial stiffness, such as pulse wave velocity, are widely utilized as surrogates for organ damage in patients with hypertension and in elderly [29]. Nevertheless, carotid CS and CSR showed a stronger connection with cardiovascular events [30].

Regarding the Kaplan-Meier survival curve cutoff values for MACE in MS patients (carotid artery CS ≤2.9% and CSR ≤0.35 $s^{-1}$), we do not have a study to compare, but the results are confirmed by those published recently regarding the connection between carotid deformation and stroke in hypertensive individuals [27].

The association between carotid strain and ischemic stroke has been demonstrated in hypertensive and diabetic patients [31, 32]. The findings regarding its association with coronary artery events remain unclear [33].

In our study that included MS patients, this population having a higher cardiovascular risk than those with hypertension or glucose metabolism abnormalities alone, carotid strain and strain rate were shown to have predictive significance for both ischemic atherothrombotic stroke and acute coronary events.

## 5. Limitations

A limitation of this study, as of other studies involving carotid IMT and carotid plaque measurements, is the inter-observer and intra-observer reproducibility of measures [34]. Data reproductibility must be assured.

The population number included is relatively low, as is the event number. Additional large-scale population studies are required to corroborate these results.

## 6. Conclusions

Carotid CS and CSR were independent predictors of major cardio- and cerebro-vascular events in prospectively monitored MS patients without established cardiovascular disease. Carotid deformation could be valuable as an early prognostic indicator for the cardiovascular risk in this population group.
